# Insights into long noncoding RNAs of naked mole rat (*Heterocephalus glaber*) and their potential association with cancer resistance

**DOI:** 10.1186/s13072-016-0101-5

**Published:** 2016-11-10

**Authors:** Jian-Jun Jiang, Le-Hua Cheng, Huan Wu, Yong-Han He, Qing-Peng Kong

**Affiliations:** 1 State Key Laboratory of Genetic Resources and Evolution, Kunming Institute of Zoology, the Chinese Academy of Sciences, Kunming, 650223 China; 2 Kunming College of Life Science, University of Chinese Academy of Sciences, Beijing, 100049 China; 3 The School of Life Science, University of Science and Technology of China, Hefei, 230027 China

**Keywords:** Naked mole rat, Long noncoding RNA, Expression profiles, Coexpression, Cancer resistance

## Abstract

**Background:**

Long noncoding RNAs (lncRNAs) are a class of ubiquitous noncoding RNAs and have been found to act as tumor suppressors or oncogenes, which dramatically altered our understanding of cancer. Naked mole rat (NMR, *Heterocephalus glaber*) is an exceptionally long-lived and cancer-resistant rodent; however, whether lncRNAs play roles in cancer resistance in this seductive species remains unknown.

**Results:**

In this study, we developed a pipeline and identified a total of 4422 lncRNAs across the NMR genome based on 12 published transcriptomes. Systematic analysis revealed that NMR lncRNAs share many common characteristics with other vertebrate species, such as tissue specificity and low expression. BLASTN against with 1057 human cancer-related lncRNAs showed that only 5 NMR lncRNAs displayed homology, demonstrating the low sequence conservation between NMR lncRNAs and human cancer-related lncRNAs. Further correlation analysis of lncRNAs and protein-coding genes indicated that a total of 1295 lncRNAs were intensively coexpressed (*r* ≥ 0.9 or *r* ≤ −0.9, *cP* value ≤ 0.01) with potential tumor-suppressor genes in NMR, and 194 lncRNAs exhibited strong correlation (*r* ≥ 0.8 or *r* ≤ −0.8, *cP* value ≤ 0.01) with four high-molecular-mass hyaluronan related genes that were previously identified to play key roles in cancer resistance of NMR.

**Conclusion:**

In this study, we provide the first comprehensive genome-wide analysis of NMR lncRNAs and their possible associations with cancer resistance. Our results suggest that lncRNAs may have important effects on anticancer mechanism in NMR.

**Electronic supplementary material:**

The online version of this article (doi:10.1186/s13072-016-0101-5) contains supplementary material, which is available to authorized users.

## Background

Naked mole rats (NMRs; *Heterocephalus glaber*) are small, nearly hairless, subterranean rodents that are renowned for their longevity and cancer resistance [1, 2]. In comparison with a similarly sized house mouse, the NMR exhibits unusual longevity, with a maximum lifespan exceeding 30 years, making NMR the longest-living rodent [3–5]. In addition, NMRs are able to maintain health until almost the end of their lives and displayed exceptional resistance to multiple age-related diseases, such as cancer [6]. The high-molecular-mass hyaluronan (HMM-HA) was proved to play a key role in regulating the cancer resistance in NMR [7]. In the NMR cells, tumor resistance is mediated by signals from the HMM-HA triggering the induction of *INK4* (inhibitors of cyclin-dependent kinase 4) locus expression. The *INK4* locus encodes a novel product named pALT^INK4a/b^ which may have a crucial contribution to tumor resistance and longevity of NMR [8]. A recent study suggested that NMR-specific alternative reading frame (*ARF*) and the disruption of oncogene embryonic cell-expressed Ras (*ERAS*) regulate tumor resistance in NMR-induced pluripotent stem cells (NMR-iPSCs) [9]. However, the deeper mechanisms of anticancer in NMRs are not well understood, which pushes forward us to look for a better understanding of the ability of cancer resistance in NMR.

Long noncoding RNAs (lncRNAs) are operationally defined as RNA transcripts longer than 200 bp that do not appear to have coding potential [10]. Recent studies indicated that lncRNAs have become new players in cancer [11] and attracted plenty of attention in scientific community due to their altered expressions and dysregulated functions as tumor suppressors or oncogenes in various human cancer types [12, 13]. Therefore, a preferable comprehending of lncRNAs becomes important and essential for cancer research. With the rapid development of next-generation sequencing (NGS) techniques and in silico analysis, large sets of lncRNAs have been identified in many species; however, it has yet to be applied in NMR. Considering the close connections between lncRNAs and cancers, it becomes urgent and necessary to identify and feature lncRNAs in the attractive cancer-resistant rodent, NMR.

In this study, we characterized for the first time the genome-wide lncRNA profiles in NMR and identified the association of lncRNAs with tumor-associated genes. Our results provide new insights into the longevity and cancer resistance in NMR, which is of essential significance in enhancing our understanding of cancer and especially broaden our knowledge on mechanisms of cancer resistance.

## Results

### Genome-wide identification and characterization of NMR lncRNAs

**Fig. 1:**
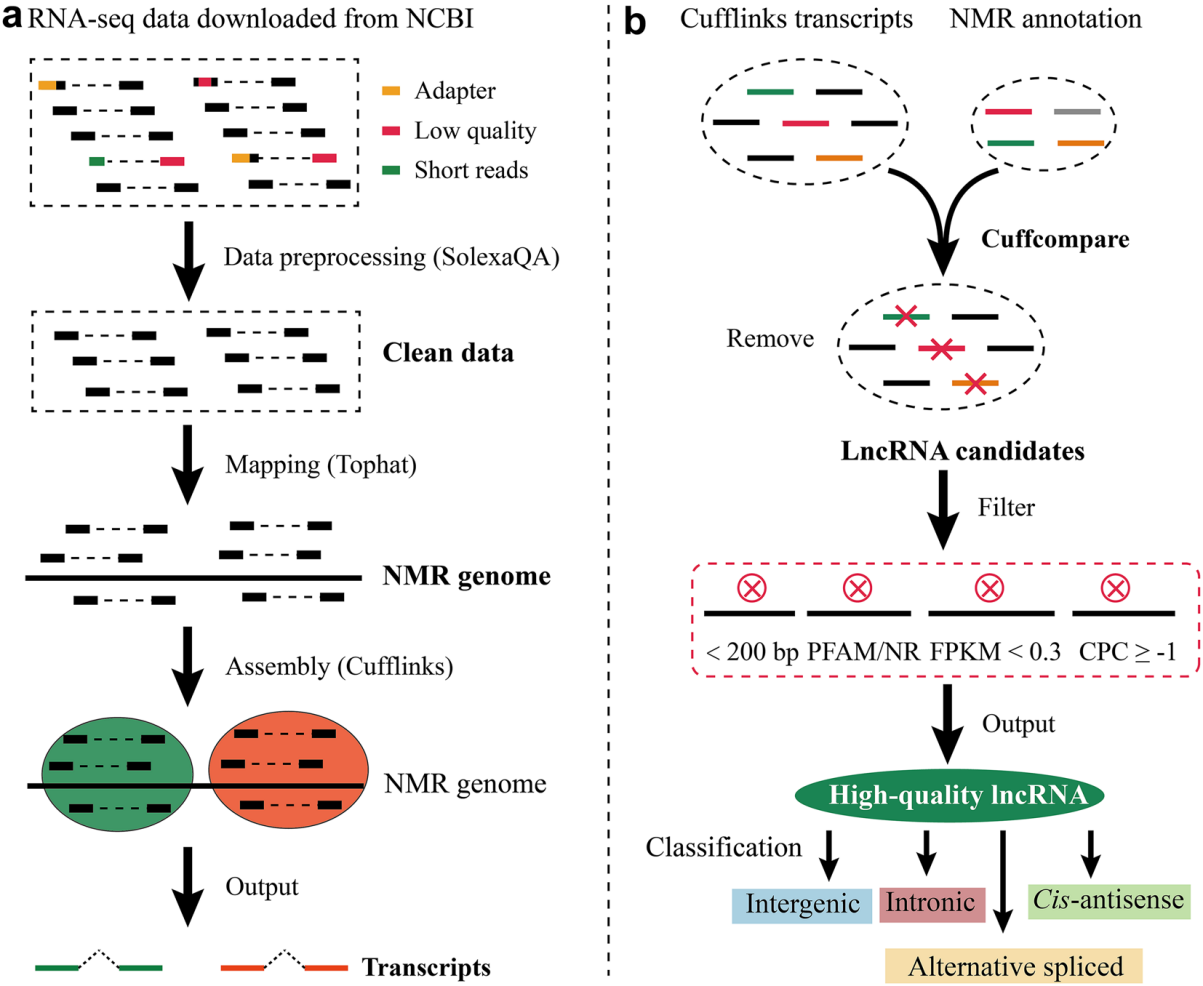
Pipeline used for identifying lncRNA in naked mole rat. **a** Data preprocessing and transcriptome assembly. **b** LncRNA identification and classifications

**Fig. 2:**
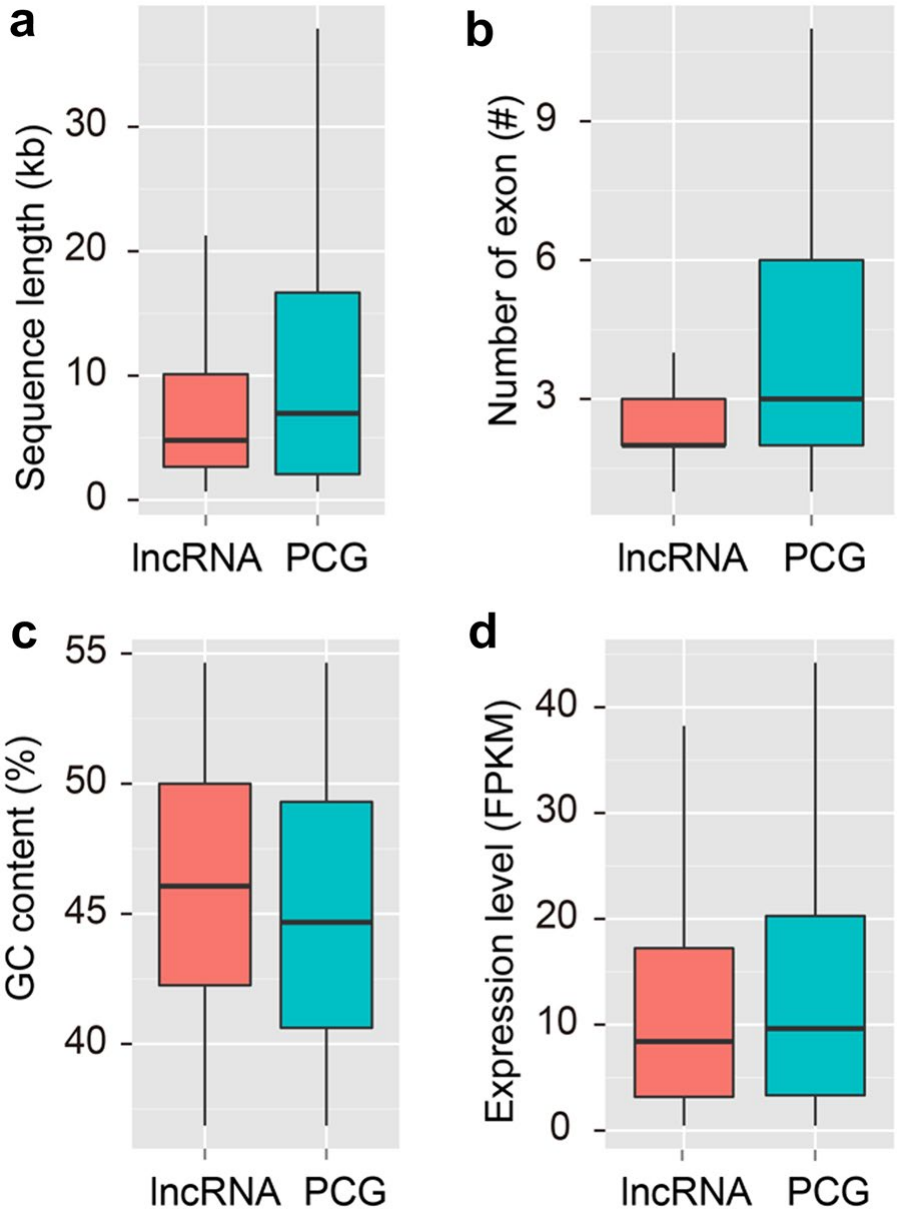
Comparisons between lncRNAs and protein-coding genes (PCGs) in naked mole rats. **a** Sequence length. **b** Number of exon. **c** GC content. **d** Expression level

In order to comprehensively identify NMR lncRNAs, 12 transcriptomes of three tissues (kidney, liver, and brain) from newborn, 4-year-old, 4-year-old with low-oxygen-treated and 20-year-old NMRs were collected [3] (Additional file 1: Table S1). Raw datasets were first subjected to SolexaQA (-h 20, -l 30) [14] to remove low-quality and short reads, and 67.8 million reads were retained for further analysis. Using a stringent filtering pipeline (Fig. 1), 4422 potential lncRNAs yielded, consisting of 3684 long intergenic noncoding RNAs (lincRNAs), 733 antisense lncRNAs (anti-lncRNA) and 5 lncRNAs transcripted from intronic regions (in-lncRNA) (Table 1). The average length of NMR lncRNAs is 16,625 bp, of which in-lncRNAs have a significant shorter length (3922 bp) than anti-lncRNA (19,668 bp) and lincRNA (16,037 bp). Compared with protein-coding genes (PCGs), lncRNAs exhibited a shorter length (Fig. 2a), less average number of exons per lncRNA transcript (2.51 vs. 8.05, Fig. 2b), but higher GC content (Fig. 2c). As expected, lncRNAs showed a significant lower expression level than PCGs by comparing the fragments per kilobase of exon per million fragments mapped (FPKM) values (Fig. 2d), which has also been observed in other species such as rainbow trout [15] and pacific oyster [16]. In conclusion, lncRNAs in NMR displayed higher GC content but shorter length, less exon numbers and lower expression level in comparison with PCGs (Fig. 2).

**Table 1:** High-quality lncRNAs identified in naked mole rat

	Number	Avg length (bp)	Exon number	Avg ExpLev^a^
Intergenic	3684	16,037	2.51	33.74
Intronic	5	3922	1.00	9.31
Antisense	733	19,668	2.55	7.50
Total	4422	16,625	2.51	29.33

^a^FPKM value was estimated by TopHat and Cufflinks with default parameters; *Avg* Average

### Expression profiles of NMR lncRNAs

**Fig. 3:**
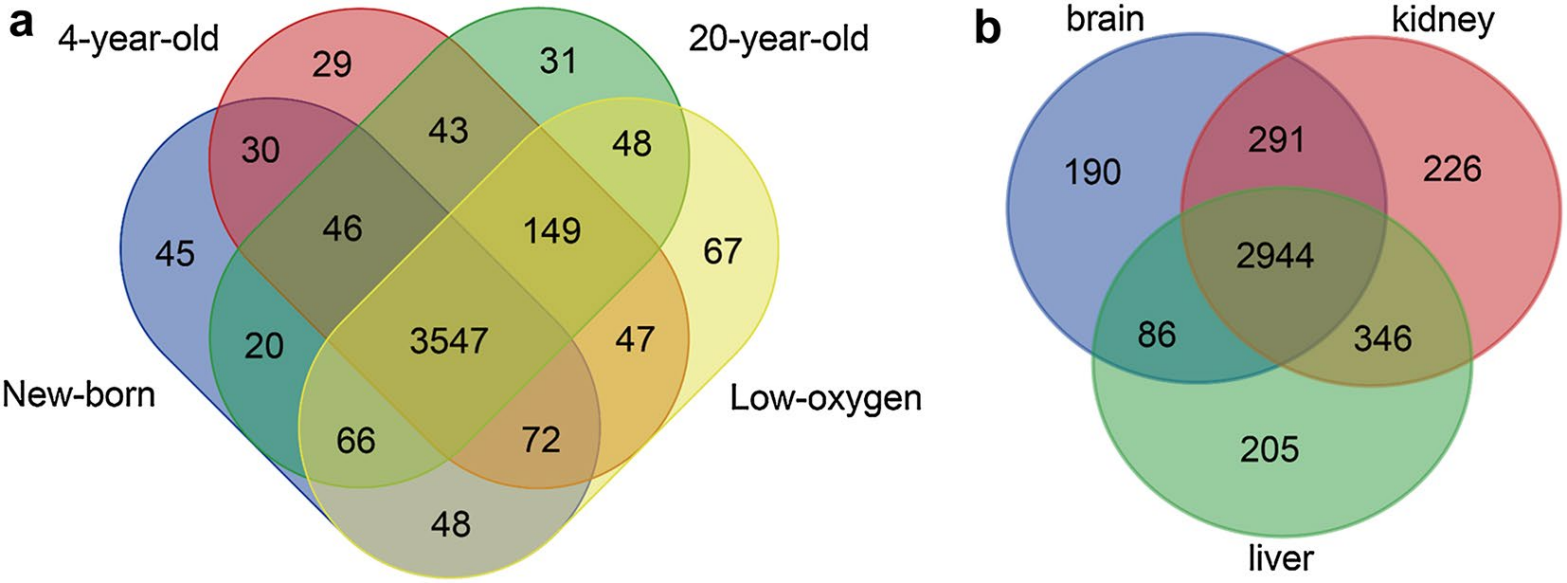
Distribution of naked mole rat lncRNAs in different tissues at different ages and tissues. **a** Ages. **b** Tissues

RNA-seq datasets from newborn, 4 year-old and 20-year-old NMRs were obtained to characterize the expression pattern of the lncRNAs. More than 80% of lncRNAs are expressed at all ages of NMR. There are 45 lncRNAs, 29 lncRNAs and 31 lncRNAs specifically expressed in newborn, 4-year-old and 20-year-old NMRs, respectively. The age-specific expression of lncRNAs indicates that some lncRNAs may play roles in the growth and development of NMR. In addition, we found 67 lncRNAs expressed particularly in 4-year-old NMR with low-oxygen treatment, suggesting them to be likely involved in low-oxygen metabolism (Fig. 3a, Table Additional file 2: S2).

Tissue-specific expression of lncRNAs was investigated using RNA-seq datasets from NMR livers, kidneys and brains. Consistent with other vertebrate species [17, 18], NMR lncRNAs displayed a significant tissue-specific expression pattern. 3667 lncRNAs are expressed at least in one tissue type, 190 are expressed merely in brains, 226 specifically in kidneys, and 205 in livers (Fig. 3b, Additional file 2: Table S2).

### Differential expression of lncRNAs across developmental tissues

**Fig. 4:**
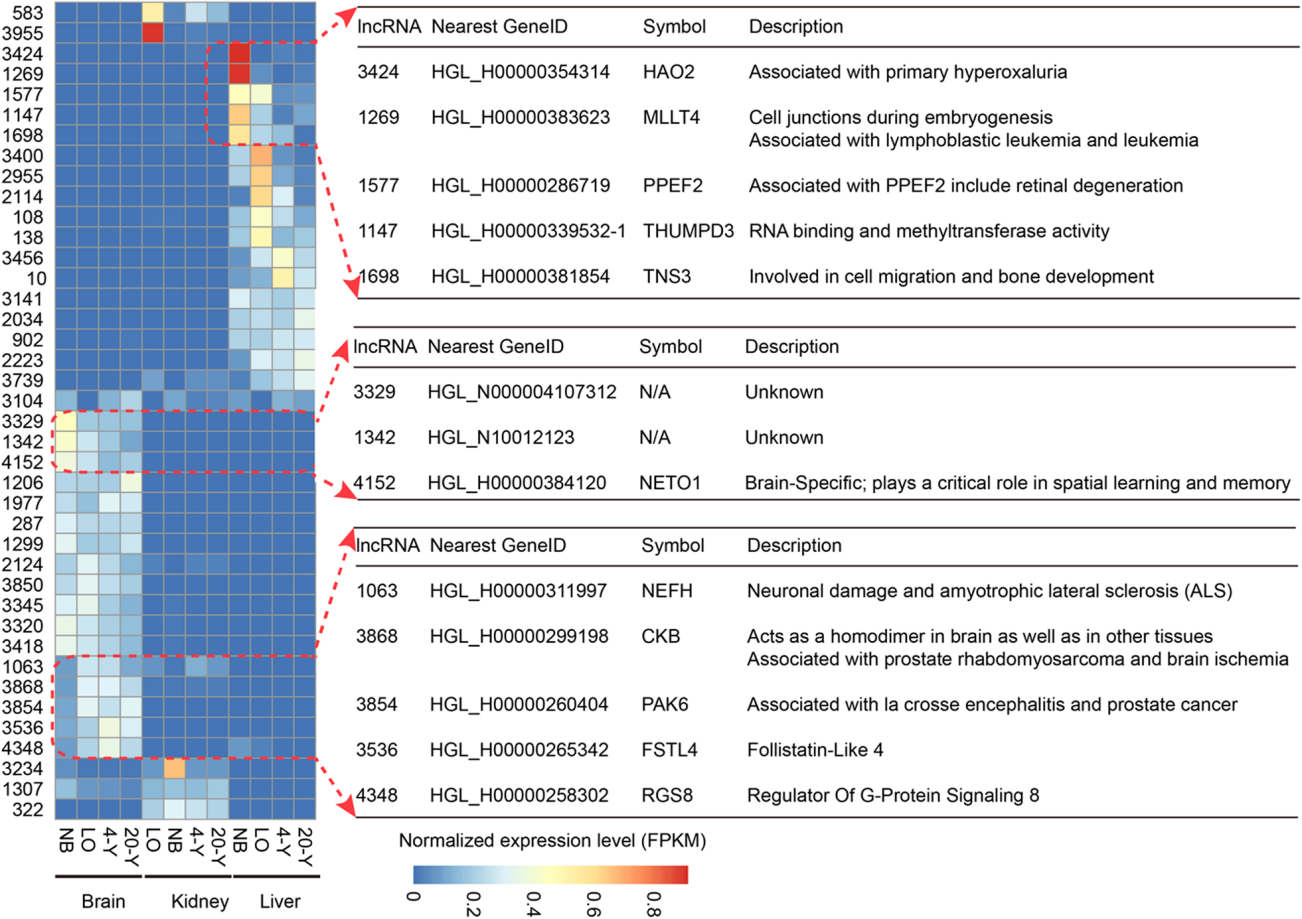
Differential expression of lncRNAs across developmental tissues. Heatmap indicates the differential expression of 40 lncRNAs in brain, kidney and liver tissues of naked mole rats (*left panel*), and the function descriptions of 13 PCGs near lncRNAs of interest are showed (*right panel*)

We next utilized these 12 RNA-seq datasets to explore the expression dynamics of lncRNAs in the NMR genome. A total of 40 lncRNAs were found differentially expressed across the developmental tissues with a fold change >2 and false discovery rate (FDR) < 0.05 (Fig. 4, Additional file 3: Figure S1, Additional file 4: Table S3). Recent studies indicated that transcription of ncRNAs including some lncRNAs may act to regulate the capacities of their chromosomal neighboring PCGs, both negatively and positively [19, 20]. Therefore, we selected 13 lncRNAs whose expressions significantly varied in livers or brains and acquired their nearest neighboring PCGs for further analysis (Fig. 4). The 13 lncRNAs are likely to be highly expressed in either liver or brain of the newborn (NB) NMR or in the liver of the older individuals, and we found that their nearest PCGs also exhibited metabolism-related or brain-related functions, indicating that some lncRNAs may have a functional connection with their neighboring PCGs.

### Conservation analysis between NMR, human and mouse lncRNAs

**Fig. 5:**
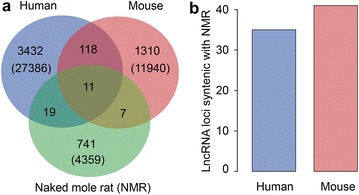
LncRNA conservation analysis among human (HUM), mouse (MUS) and naked mole rat (NMR). **a** Sequence conservation. **b** Positional conservation

To identify homologs of the NMR lncRNAs in humans and mice, lncRNA sequences of two species were downloaded from GENCODE database. In all 27, 817 lncRNAs of humans (version 23) and 12,169 of mice (version M7) were requested. Orthologous analysis of lncRNAs of three species was performed using OrthoMCL with BLASTN program [21], which identified more than 4359 (98%) NMR lncRNAs with no detectable homologs in both humans and mice lncRNA (Fig. 5a). Finally, merely 11 orthologous groups are retained across three species, revealing the deficiency of sequence conservation in lncRNAs (Additional file 5: Table S4).

LncRNAs display higher conservation in genomic position than sequence in diverse organisms [18, 22, 23]. Therefore, we conducted the analysis of lncRNA positional conservation among three species. As a consequence, 35 and 41 NMR lncRNAs show conservation with human and mice, respectively (Fig. 5b).

### Homologous analysis of NMR lncRNA and human cancer-related lncRNAs

NMR, a strictly subterranean and extraordinarily long-lived eusocial mammal, was found resistant to both spontaneous cancer and experimentally induced tumorigenesis [3, 24]. To investigate the sequence conservation between NMR lncRNAs and cancer-related lncRNAs, homologous analysis was performed. In this study, a total of 1057 experimentally supported lncRNAs associated with 86 human cancers were requested from Lnc2Cancer database [25], and then BLASTN [26] homology search with NMR lncRNAs, coverage of query and/or target >40% and *E*-value < 1e−5 were retained. As a result, only 5 NMR lncRNAs showed homology (Table 2), indicating low sequence conservation between NMR lncRNAs and human cancer-related lncRNAs. BLASTN searching of the 5 NMR lncRNAs that exhibited homology with human cancer-related lncRNAs (NMR-HCRLs) against to the NONCODE database [27] found highly similar sequences not only in human but also in mouse, rat, and etc. (data not shown), indicating that they might be conserved across species.

**Table 2:** Five NMR lncRNAs show homology with human cancer-related lncRNAs

LncRNA	Human	Qcov	Tcov	Iden	Cancer name
4086	ENST00000602892	40.5	60.5	89.4	Acute megakaryoblastic leukemia
3653	ENST00000540687	27.8	63.8	83.6	Hepatocellular carcinoma
172	ENST00000605417	91.7	9.8	96.3	Lung adenocarcinoma
3712	ENST00000409139	55.8	34.9	81.9	Gastric cancer/hepatocellular carcinoma
1169	ENST00000493116	92.3	73.0	90	Hepatocellular carcinoma/lung squamous cell carcinoma

*qcov* coverage of query to target, *tcov* coverage of target to query, *iden* identity

To determine PCGs in NMR that possibly correlated with the 5 NMR-HCRLs, we conducted coexpression analysis between mRNAs expressed in four or more developmental tissues and the 5 NMR-HCRLs. Consequently, 271 PCGs were intensively positively coexpressed with these 5 lncRNAs (Additional file 6: Table S5), while only 12 PCGs in NMR were negatively coexpressed with them (*r* ≥ 0.9 or *r* ≤ −0.9, *cP* value ≤ 0.01) (Additional file 6: Table S5). The 271 PCGs were further classified by Gene Ontology (GO) together with KEGG pathway analysis. As a result, we found a significant enrichment of 106 GO terms and 3 KEGG pathways including Neuroactive Ligand Receptor Interaction (*hsa04080*), Maturity Onset Diabetes of the Young (*hsa04950*) and Glycosaminoglycan Biosynthesis Heparan Sulfate (*hsa00534*) (*P* < 0.01) (Additional file 7: Table S6).

### Coexpression analysis of NMR lncRNAs with potential tumor-suppressor genes

To further explore potential associations between NMR lncRNAs and cancer resistance, human tumor-suppressor genes were utilized for further analysis. At the first step, a total of 1217 experimentally verified human tumor-suppressor genes were requested from TSGDB database [28], and then BLAST homology search with PCGs annotated from NMR genome [29]. Overall, we found that 901 PCGs (Additional file 8: Table S7) of NMR are homologs of human tumor-suppressor genes, which were considered as potential tumor-suppressor genes of NMR genome (PTSGs-NMR). In order to precisely analyze the correlationship between lncRNAs and cancer resistance, PTSGs-NMR and lncRNAs expressed in all developmental tissues were reserved for next-step analysis. As a result, 2091 lncRNAs and 726 PTSGs-NMR were retained for the ultimate coexpression analysis. Coexpression results show that ~61.93% (1295/2091) lncRNAs are intensively coexpressed with PTSGs-NMR (*r* ≥ 0.9 or *r* ≤ 0.9, *cP* value ≤ 0.01). Interestingly, more than 64% (834/1295) lncRNAs are positively coexpressed with a group of PTSGs-NMR and meanwhile exhibit negative coexpression with some other PTSGs-NMR as well, suggesting the possible role of lncRNAs in cancer resistance (Additional file 9: Table S8). To investigate the ratio of lncRNAs that coexpressed with potential tumor-suppressor genes in tumor-prone animals such as rat, we downloaded rat lncRNA sequences from NONCODE database [27] and 12 RNA-seq datasets across three tissues types (kidney, liver and brain) at four developmental stages of rats [30], then performed coexpression analysis between potential tumor-suppressor genes of rat (PTSGs-Rat) and lncRNAs using the same method and standard in NMR. The FPKM value of rat mRNA was requested from Rat body map database [30]. In all, 706 PSTGs-Rat and 16,328 lncRNAs were retained for coexpression analysis. About 60.41% (9864/16,328) lncRNAs are strikingly coexpressed with 670 tumor PSTGs-Rat which is lower than that in NMR (Additional file 10: Table S9).

### Coexpression analysis of NMR lncRNAs with four HMM-HA-related genes

HMM-HA, the powerful trigger for the early contact inhibition (ECI) observed in NMR cells [31, 32], mediates the cancer resistance in NMR [33]. A previous study revealed that the HA signaling triggering ECI in NMR is in part transmitted via the CD44 receptor which interacts with neurofibromin 2 (NF2) on the cytoplasmic face [33]. In addition, silencing of hyaluronan synthase 2 (*HAS2*) or overexpression of HA-degrading enzyme (*HYAL2*) in NMR cells led to susceptibility to malignant transformation and tumorigenesis in mice [7]. Hence, in order to comprehensively explore lncRNAs that related to HMM-HA metabolism in NMR, we selected four HMM-HA-related genes (*CD44*, *NF2*, *HYAL2* and *HAS2*) to perform coexpression analysis with NMR lncRNAs that expressed in all developmental tissues. Consequently, ~9.27% lncRNAs (194/2091) are strongly coexpressed with them (105 positively and 95 negatively, *r* ≥ 0.8 or *r* ≤ −0.8 with *cP* value ≤ 0.01) (Additional file 11: Table S10). Among the 194 correlated lncRNAs, three lncRNAs including *2462*, *2463* and *2464* are transcripted from one gene locus, and coexpression analysis showed that *2462* is negatively correlated with *HAS2* while *2464* is positively coexpressed with *HYAL2*; meanwhile, both *2463* and *2464* are negatively coexpressed with *NF2*. Similar to *2464*, another two lncRNAs (lncRNA *77* and *471*) show the same correlationship with *HYAL2* and *NF2* (Table 3). Interestingly, we found that three lncRNAs (lncRNA *691*, *852* and *2980*) are positively coexpressed with *NF2* and negatively coexpressed with *HYAL2*, displaying an opposite coexpression relationship compared with lncRNA *2464* (Table 3). In summary, we discovered 3 lncRNAs that transcripted from one gene locus and had close connections with three of the four known HA-related genes of NMR. Besides, 6 lncRNAs were found strongly coexpressed with *NF2* as well as *HYAL2*, negatively or positively. To explore the connection between HA synthesis and lncRNAs of tumor-prone rodent, we analyzed lncRNAs that coexpressed with four HA-related genes in the rat. Around 11.67% (1906/16,328) lncRNAs show strong coexpression (*r* ≥ 0.8 or *r* ≤ −0.8 with *cP* value ≤ 0.01) which is a little higher than NMR (~9.27%). Nevertheless, we did not detect any rat lncRNAs coexpressed with both *NF2* and *HYAL2* like that in NMR, but found some rat lncRNAs simultaneously coexpressed with *NF2* and *HAS2* (Additional file 12: Table S11). The different coexpression patten between lncRNAs and HA-related genes in NMR and rat may contribute to HA synthesis or tumorigenesis. Collectively, these results suggest that lncRNAs might be involved in HMMA regulation.

**Table 3:** NMR lncRNAs that coexpressed with both *NF2* and *HYAL2*

LncRNA	*NF2* (*r*)	*HYAL2* (*r*)
2464	−0.811	0.839
77	−0.804	0.944
471	−0.804	0.888
691	0.902	−0.811
852	0.839	−0.916
2980	0.825	−0.874

## Discussion

In this study, 4422 lncRNAs corresponding to 2946 loci were identified from the reported NMR RNA-seq datasets including three different tissues from newborn, young adult (4-year-old) and old adult (20-year-old) NMRs. Orthologous analysis by tool OrthoMCL indicated that most of the NMR lncRNAs have detectable homology with neither human or mouse lncRNAs, demonstrating that lncRNAs lack sequence conservation. However, five human lncRNAs which were previously reported to be cancer-related lncRNAs displayed high sequence conservation with NMR lncRNAs, especially the human lncRNA ENST00000493116 (also known as SOX2 overlapping transcript, *SOX2OT*). *SOX2OT* is a lncRNA that harbors in the intronic region of SOX2 gene which is one of the major regulators of pluripotency [34]. In human cancers, *SOX2OT* is co-upregulated with *SOX2* and *OCT4* in esophageal squamous cell carcinoma [35] and was also suggested to play key roles in the induction and/or maintenance of *SOX2* expression in breast cancer [36]. In NMR genome, *SOX2OT* has high sequence homology with lncRNA *1169* which is expressed only in brains and kidney with low-oxygen treatment but not in livers of NMR. The function of lncRNA *1169* in the NMR is not clear, but BLASTN searching of lncRNA *1169* as well as the other four NMR-HCRLs against the NONCODE database found BLAST hits on the queries, not only in human but also in mouse, rat, and etc., suggesting that they are possibly conserved across species. LncRNA evolution analysis by Hezroni et al. [18] revealed that *SOX2OT* is a bona fide highly conserved lncRNAs which further strengthened our results.

LncRNA expression displays spatiotemporal and tissue-specific characteristics [37, 38] which were also observed in NMR. We found some lncRNAs were particularly expressed in newborn, 4-year-old or 20-year-old NMR, exhibiting age-specific expression. The specific expression of 31 lncRNAs that merely detected in 20-year-old NMR implies their potential connection with aging or senescence due to the important roles of lncRNAs in senescence [39]. RNA-seq and microarray studies have identified altered lncRNAs during aging and in response to various types of senescence stimuli, such as *ANRIL* [40–42], *MIR31HG* [43], *PANDA* [44, 45] and *lincRNA*-*p21* [46, 47]. Hence, our analysis of NMR lncRNAs will provide new information for senescence study.

It was reported that functions of lncRNAs can be inferred by coexpression analysis and their genome locations [48, 49]. In NMR genome, we performed coexpression analysis of lncRNAs with PTSGs-NMR and four HA-related genes. In spite of the unknown functions of NMR lncRNAs, coexpression analysis showed ~61.93% (1295/2091) of NMR lncRNAs were intensively coexpressed with PTSGs-NMR (*r* ≥ 0.9 or *r* ≤ 0.9, *cP* value ≤ 0.01). This ratio is slightly higher than that in rat, demonstrating the potential role of lncRNAs in cancer resistance of NMR. HA was verified to be involved in regulating anticancer mechanism in NMR, and we found that three lncRNAs transcripted from one gene locus are closely related to three of the four HA-related genes, especially lncRNA *2464* which coexpressed with both *NF2* and *HYAL2*. As a result, a total of six lncRNAs that coexpressed with *NF2* as well as *HYAL2* were found. Unlike the NMR, in the rat, a tumor-prone rodent, we found some lncRNAs coexpressed with both *NF2* and *HAS2*. HYAL2 is an enzyme responsible for regulating the degrading of HMM-HA which is the powerful trigger for the contact inhibition [7, 49], while NF2 (merlin) interacts with CD44 receptor and mediates the contact inhibition [50]; both of them were verified to play crucial roles in cancer resistance of NMR. Comparing with other mammalian, NMR has2 protein has some unique amino acid changes which may be partially responsible for the NMR’s unusual function of HMM-HA [33]. LncRNAs expression in NMR and rat shows different coexpression characters with HA-related genes and highlights the potential function of lncRNAs in HMM-HA regulation and cancer resistance.

## Conclusion

In summary, we first identified and featured lncRNAs across NMR genome and our integrated analysis of NMR lncRNAs suggests the high potential of these lncRNAs in regulating cancer resistance in the NMR, therefore providing new insight into understanding human cancer biology as well as promising targets of cancer treatment and anticancer drug development.

## Methods

### Data accessibility

The raw NMR and rat transcriptome datasets are available at *National Center for Biotechnology Information* sra database (http://www.ncbi.nlm.nih.gov/sra/). The assembled genome sequences and annotation files of NMR were requested from GIGA database (http://gigadb.org/dataset/100022). LncRNA sequences of human and mouse were downloaded from GENCODE database (http://www.gencodegenes.org/). LncRNA sequences of rat were downloaded from NONCODE database [27].

### LncRNA identification pipeline

A stepwise filtering pipeline (Fig. 1) was used to identify putative lncRNAs from 12 RNA-seq datasets. (1) Low-quality (Phred score < 20) and short (length < 30 bp) reads were trimmed using SolexaQA [14]. The trimmed and size selected reads were then mapped to the NMR’s genome using Tophat [50]. (2) Aligned reads were assembled and merged by Cufflinks and Cuffmerge, and then noncoding transcripts for each sample were obtained by utilizing Cuffcompare [51]. Transcripts shorter than 200 bp were excluded as putative long noncoding RNAs which were commonly defined as transcripts with length longer than 200 bp. (3) The tool coding potential calculator (CPC) was employed to assess the protein-coding potential of a transcript, and transcripts with CPC ≥ −1 were eliminated [52]. (4) To evaluate which of the remaining transcripts contains a known protein-coding domain, transcripts are BLAST to Pfam database [53] and nonredundant protein database (NR) and those that with a Pfam or NR hit are excluded. (5) Transcripts with FPKM value lower than 0.3 were removed.

### OrthoMCL, BLAST and positional conservation analyses

According to method reported previously [23], here we adopted the OrthoMCL [21] pipeline to compare the sequence similarity of lncRNAs among NMR, mouse and human using the BLASTN program. The BLASTN hits with coverage ≥50% and *E*-value ≤ 1E−5 were retained and applied to assign putative orthologous groups using Markov Cluster (MCL) algorithm. BLASTN [26] homology search was conducted between NMR lncRNA and human cancer-related lncRNAs. LncRNAs with coverage of query and/or target >40% and *E*-value < 1e−5 were retained.

To perform positional conservation analyses, we first retrieved those lncRNAs pairs with *E*-value ≤ 1E−5 from BLAST results and regarded them as putative homologous sequences. We then constructed the syntenic blocks between NMR, mouse and human using MCScanX with default parameters [54]. When considering two orthologous protein-coding genes of G1 and G2 between naked mole rat and human, we detected lncRNAs within 815 kb of G1 in NMR and within 894 kb of G2 in human as previously suggested [18]. An lncRNA was considered to be found “upstream” of the protein-coding gene when it overlapped or ended 5′ end, and “downstream” when it overlapped or started the 3′ end of the protein-coding gene. Two lncRNAs of L1 and L2 from NMR and human were considered syntenic, if they were both upstream or both downstream of G1 and G2, with the same relative orientations. Similar standards and methods were also used to identify syntenic lncRNAs between naked mole rat and mouse.

### Coexpression analysis

To investigate the possible correlationship between lncRNA and cancer-resistant, we performed coexpression analysis in both NMR and rat by testing FPKM values of their 12 transcriptomes, respectively. Tophat and cufflinks were used to obtain FPKM values of lncRNAs and mRNAs [51]. PCGs expressed in at least four developmental tissues were retained for coexpression analysis with 5 NMR-HCRLs. In coexpression analysis between lncRNAs and candidate tumor-suppressor mRNAs/HA-related genes, lncRNAs and mRNAs that expressed in all tissues were reserved for analysis in both NMR and rat.

In this study, Pearson correlation coefficient (*r*) and correlation *P* value (*cP* value) were used to assess the coexpression relationship by an in-house matlab script. *r* ≥ 0.8 or *r* ≤ −0.8 with *cP* value ≤ 0.01 was considered as strong correlation, while *r* ≥ 0.9 or *r* ≤ −0.9 with *cP* value ≤ 0.01 was deemed as intensively correlation.

### Functional enrichment analysis

To classify protein-coding genes that correlated with the 5 NMR-HCRLs, Gene Ontology and KEGG pathway enrichment analysis was performed using the hypergeometric distribution and Bonferroni correction for multiple hypotheses testing with a cutoff *P* value of 0.01.

## Additional files

**Additional file 1: Table S1.** Summary of transcriptome sequencing data of the naked mole rat used in this study.

**Additional file 2: Table S2.** Expression levels of NMR lncRNAs among different tissues.

**Additional file 3: Figure S1.** Heatmap of expression profiles of NMR lncRNAs across 12 developmental tissues.

**Additional file 4: Table S3.** Differential expressed lncRNAs identified in naked mole rat (Heterocephalus glaber) genome.

**Additional file 5: Table S4.** List of homologous lncRNA groups between human, mouse and naked mole rat.

**Additional file 6: Table S5.** List of NMR lncRNAs that intensively coexpressed with five NMR-HCRLs.

**Additional file 7: Table S6.** Functional enrichment of PCGs that intensively coexpressed with five NMR-HCRLs.

**Additional file 8: Table S7.** List of potential tumor-suppressor genes of NMR genome (PTSGs-NMR).

**Additional file 9: Table S8.** List of NMR lncRNAs that intensively coexpressed with PTSGs-NMR.

**Additional file 10: Table S9.** List of rat lncRNAs that intensively coexpressed with PTSGs-Rat.

**Additional file 11: Table S10.** List of NMR lncRNAs that strongly coexpressed with four HA-related genes.

**Additional file 12: Table S11.** List of rat lncRNAs that strongly coexpressed with four HA-related genes.

## Abbreviations

NMR: naked mole rat
LncRNA: long noncoding RNA
ncRNA: noncoding RNA
lincRNAs: long intergenic noncoding RNAs
anti-lncRNA: antisense lncRNAs
in-lncRNA: lncRNAs transcripted from intronic regions
PCG: protein-coding gene
HMM-HA: high-molecular-mass hyaluronan
FPKM: fragments per kilobase of exon per million fragments mapped
PTSGs-NMR: potential tumor-suppressor genes of NMR genome
PTSGs-Rat: potential tumor-suppressor genes of rat
NMR-HCRLs: NMR lncRNAs that exhibited homology with human cancer-related lncRNAs
FDR: false discovery rate
CPC: coding potential calculator
