# Supplementary material for: Insights into long noncoding RNAs of naked mole rat (Heterocephalus glaber) and their potential association with cancer resistance
Source: Epigenetics Chromatin. 2016 Nov 10;9:51. doi: 10.1186/s13072-016-0101-5 (PMC5103457; doi:10.1186/s13072-016-0101-5)
Supplement: Supplementary file 1 — Additional file 1: Table S1. Summary of transcriptome sequencing data of the naked mole rat used in this study. [file 13072_2016_101_MOESM1_ESM.docx]

|  | **Raw data (Gb)** | | |  | **Clean data (Gb)** | | |  |
| --- | --- | --- | --- | --- | --- | --- | --- | --- |
|  | Brain | kidney | liver |  | Brain | kidney | liver | Total |
| New-born | 4.96 | 4.34 | 4.80 |  | 3.92 | 3.46 | 3.73 | 11.11 |
| 4-year-old | 4.81 | 4.54 | 4.91 |  | 3.81 | 3.66 | 3.93 | 11.40 |
| 20-year-old | 5.25 | 5.01 | 4.75 |  | 4.16 | 4.04 | 3.85 | 12.05 |
| Low-oxygen | 5.74 | 5.93 | 5.99 |  | 4.50 | 4.83 | 4.96 | 14.29 |
| Total | 20.76 | 19.82 | 20.45 |  | 16.39 | 15.99 | 16.47 | 48.85 |

**Table S1** Summary of transcriptome sequencing data of the naked mole rat used in this study.
