# Supplementary figures and images for: Insights into long noncoding RNAs of naked mole rat (Heterocephalus glaber) and their potential association with cancer resistance
Source: Epigenetics Chromatin. 2016 Nov 10;9:51. doi: 10.1186/s13072-016-0101-5 (PMC5103457; doi:10.1186/s13072-016-0101-5)

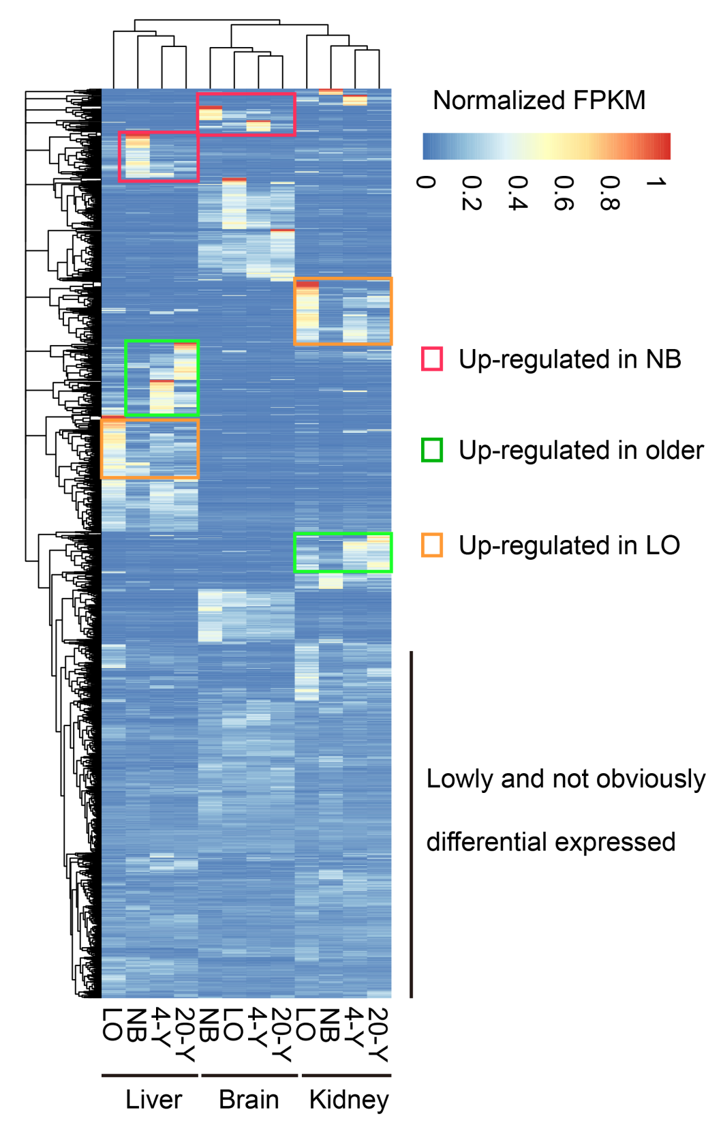

Supplement: Supplementary file 3 — Additional file 3: Figure S1. Heatmap of expression profiles of NMR lncRNAs across 12 developmental tissues. [file 13072_2016_101_MOESM3_ESM.tif]
